# Alkaline Technosol contaminated by former mining activity and its culturable autochthonous microbiota

**DOI:** 10.1016/j.chemosphere.2016.11.131

**Published:** 2017-03

**Authors:** A. Šimonovičová, P. Ferianc, H. Vojtková, D. Pangallo, P. Hanajík, L. Kraková, Z. Feketeová, S. Čerňanský, L. Okenicová, M. Žemberyová, M. Bujdoš, E. Pauditšová

**Affiliations:** a Department of Soil Science, Faculty of Natural Sciences, Comenius University, 842 15 Bratislava, Slovak Republic; b Institut of Molecular Biology, Slovak Academy of Sciences, 845 51 Bratislava, Slovak Republic; c Institute of Environmental Engineering, Faculty of Mining and Geology, VŠB – Technical University of Ostrava, Czech Republic; d Department of Environmental Ecology, Faculty of Natural Sciences, Comenius University, 842 15 Bratislava, Slovak Republic; e Department of Analytical Chemistry, Faculty of Natural Sciences, Comenius University, 842 15 Bratislava, Slovak Republic; f Institute of Laboratory Research on Geomaterials, Faculty of Natural Sciences, Comenius University, 842 15 Bratislava, Slovak Republic; g Department of Landscape Ecology, Faculty of Natural Sciences, Comenius University, 842 15 Bratislava, Slovak Republic

**Keywords:** Alkaline Technosols, Potentially toxic elements, Autochthonous isolates, Microbial biomass

## Abstract

Technosols or technogenic substrates contaminated by potentially toxic elements as a result of iron mining causes not only contamination of the surrounding ecosystem but may also lead to changes of the extent, abundance, structure and activity of soil microbial community. Microbial biomass were significantly inhibited mainly by exceeding limits of potentially toxic metals as arsenic (in the range of 343–511 mg/kg), copper (in the range of 7980–9227 mg/kg), manganese (in the range of 2417–2670 mg/kg), alkaline and strong alkaline pH conditions and minimal contents of organic nutrients. All of the 14 bacterial isolates, belonged to 4 bacterial phyla, *Actinobacteria*, *Firmicutes*; β- and γ-*Proteobacteria*. Thirteen genera and 20 species of microscopic filamentous fungi were recovered. The most frequently found species belonged to genera *Aspergillus* (*A*. *clavatus*, *A*. *niger*, *A*. *flavus*, *A*. *versicolor*, *Aspergillus* sp.) with the dominating *A*. *niger* in all samples, and *Penicillium* (*P*. *canescens*, *P*. *chrysogenum*, *P*. *spinulosum*, *Penicillium* sp.). Fungal plant pathogens occurred in all surface samples. These included *Bjerkandera adustata*, *Bionectria ochloleuca* with anamorph state *Clonostachys pseudochloleuca*, *Lewia infectoria*, *Phoma macrostoma* and *Rhizoctonia* sp.

## Highlights

• Alkaline and strongly alkaline Technosol contaminated by As, Cu, Mn, Pb, Zn caused changes in autochtonous microbiota.
• *Variovorax pantotropha* and *Staphylococcus pasteuri* Sp-12 were isolated.
• 20 fungal species belonged to 13 genera were recovered.
• The isolated microorganisms showed their high adaptation abilities.
• Different isolates can be useful for various industrial and remediation processes.

## 1 Introduction

According to World Reference Base for Soil Resources (WRB; 2006) Technosols include soils from wastes, mine spoils or ashes and they are often referred to as urban or mine soils. Such soils are formed during re-cultivation of overburdens, tailings and other spoils and wastes resulting from mining and other industrial activities (Levyk et al., 2007, Baran et al., 2014). According to Hafeez et al. (2012), Technosols could support soil functions, including primary production, but the knowledge about other ecosystemic role is limited. Mining of mineral resources results in extensive soil damage, altering microbial communities and affecting vegetation leading to destruction of environment (Sheoran et al., 2010). Mining activities, disposal of metals or metal containing material inevitable cause contamination in the surrounding ecosystem together with changes in the extent, structure and activity of soil microbial community (Ge and Zhang, 2011). Many Technosols have to be treated carefully as they may contain toxic substances resulting from industrial processes and represent severe danger. Mine soils are often physically degraded, and so they hinder plants development (Asenio et al., 2013).

Technosols at the site of Slovinky (Slovak Republic) presents fine-grained industrial material originated from iron ore mining (Petrák et al., 2011, Tóth et al., 2013). At the site, the flotation sludge was deposited during 1969–2009 reaching more than 4.8 million m^3^ upto now. The decanting plant, as high as 113 m, is the highest in Slovakia. The material contains elevated concentrations of various elements: arsenic (As), cadmium (Cd), copper (Cu), manganese (Mn), lead (Pb) and zinc (Zn) carrying potential risk in the case of leaching from substrate and being released into the surface and ground water. This mining waste site is bounded by broadleaf mixed forest with formed mostly by *Betula pendula*, *Fagus sylvatica*, *Coryllus avelanea*, *Larix decidua* and *Picea abies*. The substrate surface is occasionally moss-grown (Krokusová, 2005). Such a substrate clearly represents an extreme environment for microorganisms (Roadcap et al., 2006, Levyk et al., 2007, Wang et al., 2011, Gostinčar and Turk, 2012, Gostinčar et al., 2012, Šimonovičová et al., 2013a, Šimonovičová et al., 2013b, Šimonovičová et al., 2016). Anyway, those are well-adapted to specific conditions at the level of their micromorphological structures and metabolic activities as well.

The aim of this study was to characterize Technosol from former mining site contaminated by high amounts of arsenic and other potentially toxic elements, such as Cd, Cu, Mn, Pb, Zn, and minimal contents of organic nutrients. The aim was also to show the ability of its culturable autochthonous bacteria and microscopic fungi to grow and produce biomass despite of exceeding metal contamination and lack of organic nutrients of this particular substrate. All organisms living in Technosol showed their high adaptation abilities to stress conditions, which allow them not only to survive but to build a basis for future settlements of other organisms. In addition, it would be possible to select interesting microorganisms able to be used in different biotechnology applications such as bioleaching and bioremediation.

## 2 Material and methods

### 2.1 Study sites and sampling

**Fig. 1:**
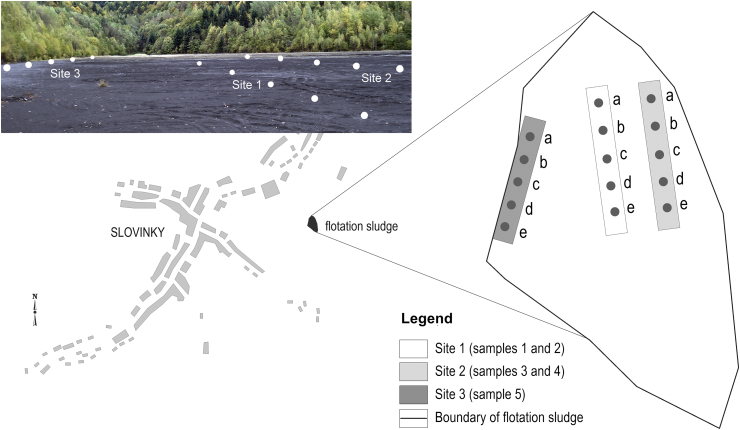
Location of the sampling sites.

**Fig. 2:**
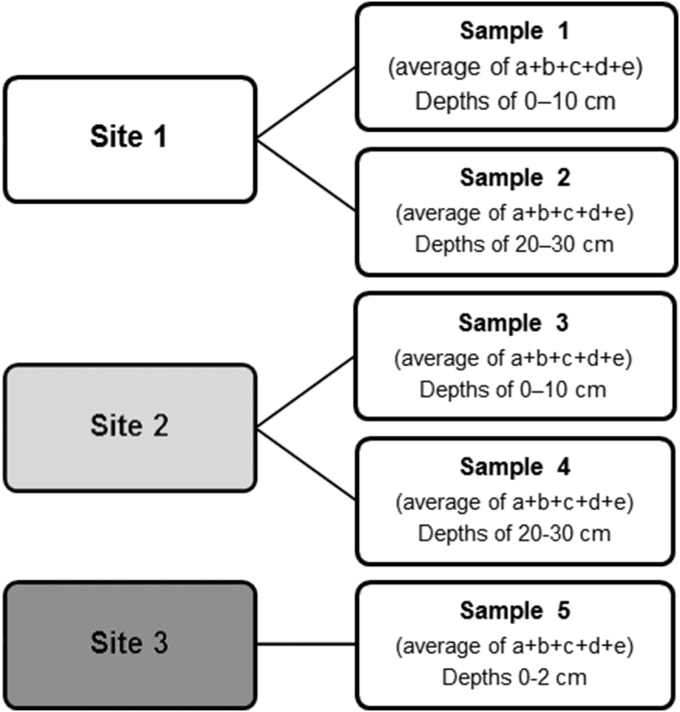
Scheme of investigated samples.

The samples were picked up from the places evidenced in Fig. 1. Samples of substrate were collected in the middle of the decanting plant (site 1) from the depths of 0–10 cm (sample 1a–e) and 20–30 cm (sample 2a–e) and in marginal parts nearby the birch bush and high grasses (site 2) from the same depths (samples 3a–e and 4a–e), respectively. The substrate from the depth of 0–2 cm (site 3, sample 5a–e), was collected below the crust of root system of mosses (Fig. 2). The humidity of soil samples was measured. Each samples had five replicates; in laboratory all samples were homogenized by quartering, passed through a 2 mm sieve and stored at 4 °C in darkness until all microbiological analyses were performed.

### 2.2 Basic chemical analyses

Values of pH_H2O_ and pH_KCl_ were measured potentiometrically and %C_ox_ (oxidizable organic carbon) content was determined by oxidimetry under laboratory conditions. The value of humus content was calculated from %C_ox_ multiplied by a conversion factor of 1.724 (Hrivňáková et al., 2011). Soil microbial biomass carbon (C_mic_) was detected by fumigation extraction method according to Jenkinson and Ladd (1981). Soil respiration, it means the amount of carbon dioxide measured according to Schinner et al. (1993) as basal respiration (B-CO_2_) in the original sample and as potential respiration (P-CO_2_) with addition of 1% glucose in the same sample. There were three replicated runs for each analytical determination and average values are showed as results.

### 2.3 Isolation and cultivation of bacteria and microscopic filamentous fungi

Cultivable soil microorganisms were determined using the method of direct plate inoculation. Bacteria were isolated Nutrient Agar w/1% Peptone, Nutrient Agar w/NaCl, Nutrient Agar No. 2, Tryptone Soya Agar, *Pseudomonas* Agar and Kligler Iron Agar (HiMedia, Mumbai, India) using the dilution of 10^4^ CFU (Colony Forming Units) per 1 g of dry sample. Inoculated agar plates were cultivated in the dark at the temperature of 30 °C for 5 days.

Microscopic fungi were isolated using the same dilution 10^4^ CFU inoculated on Potato Dextrose Agar (PDA), Malt Extract Agar (MEA) and Sabouraud Dextrose Agar (SDA; HiMedia). Inoculated agar plates were cultivated in the dark at the temperature of 25 °C for 7–10 days. All morphologically distinctive colonies were selected and purified, the isolates were maintained onto the original isolation agar media.

### 2.4 DNA extraction

Bacterial DNA was isolated using the DNeasy purification kit (Qiagen, Hilden, Germany) according to the manufacturer's instructions. The resulting high-molecular-weight DNA was stored at −20 °C and used subsequently as a template in appropriate PCR experiments.

The fungal isolates were inoculated in Sabouraud broth at 28 °C until growth. Later the fungal pellets were separated from the broth by filtration with sterile filter paper and then the DNA was extracted with DNeasy Blood and Tissue Kit (Qiagen, Hilden, Germany) following the provided protocol for animal tissue (DNeasy Tissue Kit Handbook, July 2006).

### 2.5 Bacterial ARDRA clustering and 16S rRNA sequencing identification

The bacterial isolates were clustered using amplified rDNA restriction analysis (ARDRA) and then at least one bacterial representative of each ARDRA profile were identified by 16S rRNA sequencing.

The 16S rRNA gene was amplified with primers (27F: 5′ AGAGTTTGATCCTGGCTCAG 3′, and 685R: 5′ TCTACGCATTTCACCGCTAC 3′; positions 8–27 and 704–685 in the *Escherichia coli* K12 [NC_000913] numbering system (Lane, 1991),). Each 50 μL reaction mixture contained 2 μL of the DNA template, 5 μL 10 × Taq buffer (Qiagen), 2.5 U *Taq* DNA polymerase (Hot-Star; Qiagen), 1.5 mM MgCl_2_, 200 μM dNTPs and 0.5 μM of each primer. PCRs were performed in a T1 thermal cycler (Biometra, Goettingen, Germany) with the following cycling conditions: 2 min of denaturation at 94 °C, 25 cycles of 1 min at 53 °C, 1.5 min at 72 °C, 1 min at 94 °C, and a final cycle of extension at 72 °C for 5 min. PCR products were separated by electrophoresis in a 1% (w/v) agarose gel (Merck, Germany), stained with Gold View Nucleic Acid Stain (SBS Genetech, Beijing, China). DNA bands, approximately 696 bp in size for the 16S rRNA were excised and purified using the QIAquick Gel Extraction Kit (Qiagen) according to the manufacturer's instructions.

For ARDRA analysis purified amplicons were digested separately with two endonucleases, *Alu*I and *Bst*II, and fragments were separated by electrophoresis in 1% agarose gels (w/v) (Merck, Germany), and stained with Gold View Nucleic Acid Stain (SBS Genetech). Subsamples of purified 16S rRNA amplicons (of different ARDRA patterns) were sequenced by the outsourcing facility GATC Biotech (Konstanz, Germany).

### 2.6 Amplification of fungal ITS fragment

Filamentous fungi were identified by the amplification and sequencing of the internal transcribed spacer (ITS) fragment using the primers ITS1 (TCCGTAGGTGAACCTGCGG) and ITS4 (TCCTCCGCTTATTGATATGC) (White et al., 1990). The PCR mixture (25 μl) contained 50 pmol of each primer, 2.5 mmol l^−1^ MgCl_2_, 200 μmol l^−1^ of dNTP, 1.5 U HotStar Taq plus DNA polymerase (Qiagen) and 1 × PCR buffer and 3 μl of extracted DNA. The temperature program consisted of initial denaturation at 95 °C for 5 min, 35 cycles (94 °C for 45 s, 54 °C for 1 min, 72 °C for 1 min) and a final polymerization step at 72 °C for 10 min.

The PCR products were purified using ExoSAP-IT digestion (Affymetrix, Cleveland, Ohio, USA) and sequenced for both strands by a commercial facility (GATC Biotech).

### 2.7 Bacterial and fungal strains identification and phylogenetic analysis

Specific bacterial 16S rRNA (16S rDNA) and fungal ITS sequences were edited by Chromas Lite software (version 2.01) for further DNA analysis. A Basic Local Alignment Search Tool (BLAST) search of the National Center for Biotechnology Information (NCBI) genome database (http://blast.ncbi.nlm.nih.gov/Blast.cgi) was conducted to identify sequences with highest similarity. Multiple sequence alignments and phylogenetic trees were constructed with the MEGA software (version 5.1, Tamura et al., 2011). Maximum likelihood method with 100 bootstrap replications was chosen with Tamura-Nei model of substitutions and the resulting tree was presented at the Tree Explorer of the MEGA package.

### 2.8 Nucleotide sequence accession numbers

The sequences generated in this study were deposited in the GenBank database under accession numbers from [JQ965935] to [JQ965938] and from [KC817534] to [KC817538] for bacterial 16S rRNA (16S rDNA) genes.

### 2.9 Determination of total content of selected elements

Total content of the elements Cd, Cu, Mn, Pb and Zn in the substrates was determined after decomposition with HNO_3_, HF and HClO_4_, the procedure published by Žemberyová et al. (2010). A Perkin-Elmer flame atomic absorption spectrometer 1100 B (Perkin-Elmer, USA) with air-acetylene flame (acetylene flow rate of 2.5 L min^−1^ and air flow rate of 8.0 L min^−1^) was used for heavy metal determinations (Žemberyová et al., 2014).

### 2.10 Arsenic determination

Soil sample (0.500 g) was digested in 5 mL of concentrated HNO_3_ in stainless-steel coated polytetrafluoroethylene (PTFE) pressure bomb at 160 °C in an electric oven for 6 h. The cool digest was transferred into a 50 mL volumetric flask and mixed. Arsenic was determined using ICP-OES. ICP spectrometer Jobin Yvon 70 Plus (France) was equipped with concentric nebulizer (Meinhard, USA) and cyclonic spray chamber. Used wavelength: 193.690 nm, plasma power: 1000 W.

## 3 Results and discussion

All studied substrates were classified as Technosol (WRB, 2006). The samples were strongly alkaline (pH value 8.6 for the samples 1, 3 and 5) and very strongly alkaline (pH values 9.4 and 9.1 for the samples 2 and 4 respectively). The content of organic matter (% C_ox_ and % of humus) depended on the location and the depth of sampling with the highest value of 1.38 in the sample 1 from the depth of 0–10 cm and the lowest value of 0.52 in the sample 2 from the depth of 15–30 cm. Surprisingly, the values of actual humidity were the highest in the samples from the depths of 0–2 and 0–10 cm (samples 1, 3 and 5) not from the depths 20–30 cm as it could be expected (Table 1).

The contents of all observed chemical elements highly exceeded limit values (Table 2). The highest contents of arsenic were recorded in samples 1 and 2 collected from the middle of the decanting plant. In the samples 3–5, the highest contents of cadmium, lead and zinc were detected. The contents of copper and manganese were almost the same in all the samples. The highest contents of copper, manganese, lead and zinc were determined in the sample 4 from the depth of 20–30 cm. The different concentrations of potentially toxic elements in each studied samples are very interesting because of this Technosol is a homogenous substrate of fine-grained industrial material with no significant differences in chemical elements' composition.

Isolated bacteria were identified according to their nearest (bacterial) relatives using phylogenetic analysis (Table 3). A total of 14 bacterial isolates represented 9 species or genera, respectively, belonging to 4 bacterial phyla, *Actinobacteria*, *Firmicutes*; β- and γ-*Proteobacteria*. The phylum *Actinobacteria* included 4 isolates assigned to 3 species; one isolate, MR-2 [JQ965936], to *Arthrobacter* sp. A8, one, MR-3 [JQ965937], to *Kocuria* sp. TMT4-12-2, and remaining two isolates, SL-11 [KC817538], Uncultured bacterium clone ELU0126-T312-S-NI-000133. The phylum *Firmicutes* was represented by 6 isolates of 4 species; two isolates, SL-1 [KC817534], belonged to *Staphylococcus pasteuri*, Sp-12, two other isolates, MR-4 [JQ965938], were assigned to *Bacillus* sp. BA-113, one, MR-1 [JQ965935], to *Bacillus* sp. BA-27, and the remaining one, SL-6 [KC817538], to Uncultured bacterium clone FWB10-MS. The phylum β-*Proteobacteria* comprises three our isolates, SL-5 [KC817536], of *Variovorax paradoxus*, DSM 30162, and just one isolate, SL-2 [KC817535], is taxonomically located at the phylum *γ-Proteobacteria*. In addition, 2 isolates, SL-11 [KC817538], assigned to *Actinobacteria* and one, SL-6 [KC817538], assigned to *Firmicutes*, which were first given as uncultivated bacteria, recently seem to belong to new species or genera: two of them to the phylum *Actinobacteria* and the remaining one to *Firmicutes*. Furthermore, one representative of γ-*Proteobacteria* was isolated from the sample 1, three isolates of *Actinobacteria* as well as *Firmicutes* from the sample 2, one representative of *Actinobacteria*, another one of *Firmicutes* and three isolates of β-*Proteobacteria* from the sample 3, and remaining two representatives of *Firmicutes* from the sample 5. And in the end, the only isolates identified as *Staphylococcus pasteuri*, Sp-12 and isolates identified as Uncultured bacterium clone ELU0126-T312-S-NI-000133 were originated from the samples 3 and 5 or 2 and 3, respectively. *Bacillus* and *Arthrobacter* species have been found so far in extremely alkaline (pH up to 12.8) environments (Kanekar et al., 2002, Muntyan et al., 2005), in river sediments affected by the proximity of a petrochemical industrial site (Narancic et al., 2012) and so on. Species of genus *Kocuria* might be isolated from various environments such as alkaline (pH 11.4) groundwater (Tiago et al., 2004) and from different habitats in Antarctica (Satyanarayana et al., 2005). The species diversity of *Kocuria* bacteria suggests their capacity to adapt and prosper in their own ecological niche.

The isolation of bacteria belonged to the genus *Pseudomonas* was not surprising because the nutrient demands of pseudomonads are very modest. Generally *Pseudomonas* strains can colonize various natural and extreme ecosystems, including highly contaminated soils, waters and sediments (Otenio et al., 2005, Rastogi et al., 2009, Krishna et al., 2012, Vojtková et al., 2012, Šimonovičová et al., 2016, Alisawi et al., 2017). The strain *Pseudomonas* sp. B7 was isolated from soil samples nearby the Koyama Lake at the Tottori University, Japan in 2007. The strain possessed homocholine-degrading activity.

*Variovorax paradoxus* was isolated from the soil enriched with pantothenate as *Hydrogenomonas pantotropha* in 1969 (Davis et al., 1969). *Variovorax paradoxus*'s diverse metabolic capabilities enable it to degrade a wide array of recalcitrant organic pollutants. Both its catabolic and anabolic capabilities have been suggested for biotechnological use, such as to neutralize or degrade pollutants at contaminated sites (Nishino and Spain, 2006, Satola et al., 2013). This species was also isolated from gold-arsenopyrite mine drainage water (Piotrowska-Seget et al., 2005). Although our study confirmed the high adaptation ability of *Variovorax paradoxus* it is possible to see that our isolate can grow in the presence of higher concentrations of As, Cu, Pb, Zn (Table 2) than in the previous reports.

The occurrence of the *Staphylococcus pasteuri* Sp-12 bacterial strain in arsenic contaminated substrates is also here presented for the first time. Up to date, species of the *Staphylococcus* genera were isolated only from e. g. acid milk products, fermented food, human urine and vomitus (Chesneau et al., 1993). Identified clone Sp-12 was found as a part of biodiversity of lactic acid bacteria from fermented vegetable and its sequence is deposited in the GenBank since 2010. The identification of bacteria *S. pasteuri* in fresh sausages (Rantsiou et al., 2005) is interesting because there is an unconfirmed metabolic relation of this genus to toxic and carcinogenic substances produced during smoking processes. The identification of the *S*. *pasteuri* Sp-12 strain under strongly alkaline conditions (samples 3 and 5) indicated its high tolerance to extreme pH values.

Due to very high contamination of substrates with arsenic, only 13 genera and 20 species of microscopic fungi were identified from all our samples (Table 4). Saprotrophic microscopic fungi include mainly species of genera *Aspergillus* (*A*. *clavatus*, *A*. *niger*, *A*. *flavus*, *A*. *versicolor*, *Aspergillus* sp.) with dominating strain *A*. *niger* in all the samples and *Penicillium* (*P*. *canescens*, *P*. *chrysogenum*, *P*. *spinulosum*, *Penicillium* sp.). Ubiquitous *Aspergillus* and *Penicillium* species were identified in different substrates such as extreme environments with heavy metal contamination (Amich et al., 2010, Ezzouhri et al., 2009, Šimonovičová et al., 2013a, Šimonovičová et al., 2013b, Rasool and Irum, 2014), biological soil crusts (Kubátová et al., 2002, Kubátová and Prášil, 2008, Bates et al., 2010, Bates et al., 2012) and they have very high potential for bioremediation (Lietão, 2009, Ren et al., 2009, Dugal and Gangawane, 2012) and can be used for heavy metal bioleaching (Anjum et al., 2010, Zeng et al., 2015). Minimal humus content is a limit factor for expansion of Mucormycotina species, except of *Mortierella alpina* in the sample 3. This species was also isolated from another exposed environment as biological soil crusts (Bates et al., 2010, Bates et al., 2012) or glacier foreland (Ali et al., 2013). On the other side, fungal pathogens were very frequent in the samples 1, 3 (from the depth of 0–10 cm) and 5 (from the depth of 0–2 cm), it means from the surface horizon (Table 1). There were species *Bjerkandera adustata*, *Bionectria ochloleuca* - the species very active in bioremediation (Kota et al., 2014) and poly(butylene succinate) degradation (Mei et al., 2012) with the anamorph state *Clonostachys pseudochloleuca*, *Lewia infectoria* (anamorph state *Alternaria infectoria*), *Myrothecium roridum*, *Phialocephala*, *Phoma macrostoma* and *Rhizoctonia* sp. Occurrence of plant pathogens in surface horizons is probably influenced by plant community structure in the vicinity of the area studied. The identification of *Phialocephala* sp. is very interesting because this genus include relatively few species, therefore this isolate could be in the future better characterized. Its isolations are recorded from various habitats such as soil, bark, wood plant growing in cool or cold environments. Based on morphological and physiological variability, *Phialocephala* spp. still represent a heterogeneous group (Jacobs et al., 2001, Jacobs et al., 2003). According to Nagai et al., 1995, Nagai et al., 1998, Kladwang et al., 2003 and Mlitan et al. (2013), many fungal strains are alkaliphilic and alkali-tolerant, e. g. *Acremonium furcatum* capable to growth very well at pH 9–10 and in the environment contaminated with Zn, Pb, Cd, Ni and Co (Iram et al., 2009, Abbas et al., 2014). From alkaline soils, species of genera *Aspergillus*, *Penicillium*, *Trichoderma*, *Mortierella alpina* and others are reported (Klich, 2002, Kubátová and Prášil, 2008). This screening demonstrates that there exists a population of microscopic filamentous fungi able to tolerate high pH values (from 8.6 to 9.4). Importantly, alkaline-tolerant fungi are also very often isolated from some acid environments (Nagai et al., 1995, López-Archilla et al., 2001, López-Archilla et al., 2004, Šimonovičová et al., 2013a, Šimonovičová et al., 2013b).

On the basis of biodiversity of microscopic fungi (Table 5), similarity and dissimilarity of mycocoenoses in % according to Jaccard (S_J_) and Sörensen (S_S_) was determined (Kosman and Leonard, 2005, Chao et al., 2006). Despite of the low values of S_J_ and S_S_, there was a certain similarity between the samples 2 and 4 (from the depth of 20–30 cm) and the samples 1 and 2 that were taken from one sampling site in two different depths. But a dissimilar ecosystem was displayed below the crust of root system of mosses (sample 5) nearby the mixed forest community.

**Fig. 3:**
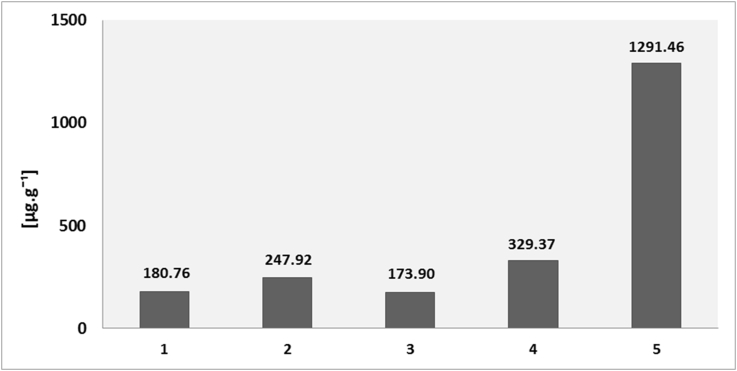
Soil microbial biomass values (C_mic_) estimated by fumigation extraction in the investigated samples of Technosol.

Values of microbial biomass (C_mic_) determined by fumigation extraction (Vance et al., 1987) were very similar in samples 1–4 and ranged from 173.90 μg C to 329.37 μg C per gram of substrate. The highest value (1291.46 μg C per gram of substrate) was observed in the sample 5 where was seven times higher than in the other samples (Fig. 3). This is probably due to moss layer typical for this site that built up more suitable microclimatic conditions for the existence of soil microorganisms (10 species of microscopic fungi were isolated). The activity of soil microbial communities is closely connected to the soil humidity, soil temperature and especially to the bioavailability of organic matter (Akmal et al., 2005, Liao et al., 2007, Asenio et al., 2013, Wright and Coleman, 2000). The lowest values of microbial biomass founded in the samples 1 and 3 pointed not only to the high content of arsenic and other toxic elements but to limited available organic substrates and nutrients through biogeochemical cycles to support soil microbial community-evolution (Wang et al., 2011).

**Fig. 4:**
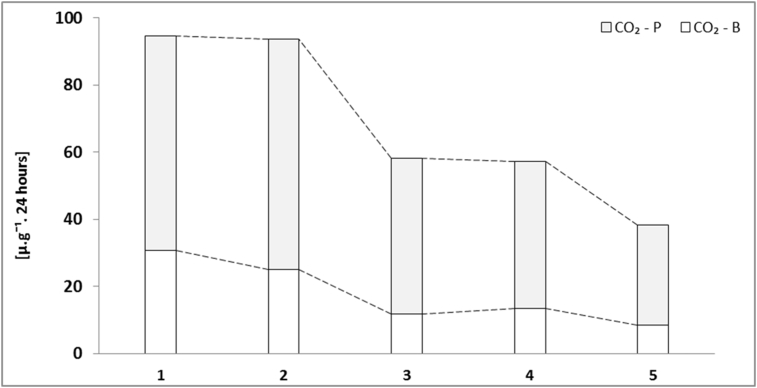
Basal and potential respiration (CO_2_ - B; CO_2_ - P) determined by quantifying the carbon dioxide released in the process of microbial activity in the investigated samples of Technosol.

The average value of basal respiration increased in this order: samples 5 < 3 < 4 < 2 and <1. However, the highest value of potential respiration was found in the sample 2 (Fig. 4). Relatively low values of the basal respiration of microorganisms (up to 31 μg/g of substrate) in all our samples were definitely caused by the high contents of potentially toxic elements. Such contents of metals resulted in decreasing of carbon dioxide production by microorganisms (Feketeová et al., 2016). Several authors (Yuangen et al., 2006, Zhang et al., 2015) suggest soil microbial indicators such as microbial biomass and basal respiration to be useful indicators of polluted environment.

According to Horikoshi (1999) microorganisms able to grow optimally or very well at pH above 9 (alkaliphiles), can be isolated from normal environments as garden soils, although, their viable counts are higher in samples from alkaline environments. The cell surface of these microorganisms plays a key role in keeping the intracellular pH value in the range between 7 and 8.5, allowing alkaliphiles to thrive in alkaline environments. Certain groups of microscopic fungi have evolved specialized mechanisms that enable them to resist even the most extreme environmental condition (Gostinčar and Turk, 2012, Gostinčar et al., 2012). Long-term effect of the extreme environmental conditions combined with the presence of heavy metal ions cause mutations expressed in macromorphological and micromorphological features of microorganisms. Ezzouhri et al. (2009) and Šimonovičová et al., 2013a, Šimonovičová et al., 2013b showed that both these factors influenced gene expression, metabolism and consequently the morphological appearance in *Aspergillus niger* strains. Presence of living mutants of *A. niger* strains in the extreme environments indicates the adaptation of fungi from the point of long-term effect.

## 4 Conclusions

Arsenic Technosols, from alkaline to strongly alkaline, contaminated by potentially toxic elements caused the changes of the structure of culturable microbial community, its biomass and activity. Microbial biomass depended to the availability of organic substrates, nutrients and through the biogeochemical cycles. Microorganisms were significantly inhibited mainly by exceeding levels of potentially toxic metals, such as Cd, Mn, Pb and Zn, minimal contents of organic nutrients, soil humidity and pH. This study confirmed that all bacterial and fungal isolated were capable to grow under very high contents of As, Cu, Pb and Zn. Moreover, it is evident that the microbiome of investigated samples is strictly related to the chemical characteristics.

*Variovorax paradoxus* was efficiently adapted to such high contents of metal(loid)s. Aspergilli were the most isolated microscopic fungi and among them *A. niger* was the dominant species. In addition, in all samples from the surface, phyto-pathogenic microscopic fungi were also frequently identified. The microorganisms isolated in our study showed their importance in biogeochemical cycles of potential toxic elements, furthermore their adaptation to these extreme conditions can be applied to different industrial and remediation processes.

## Conflict of interest

The authors declare that they have no conflict of interest.

## Figures and Tables

**Table 1:** Selected characteristic of investigated samples of Technosol.

Sample	Depth (cm)	pH		% C_ox_	% of humus	Actual humidity (%)
		H_2_O	KCl			
1	0–10	8.6	8.3	0.8	1.38	21.0
2	20–30	9.4	9.1	0.3	0.52	16.0
3	0–10	8.6	8.3	0.6	1.03	32.8
4	20–30	9.1	8.9	0.7	1.21	26.3
5	0–2	8.6	8.2	0.6	1.03	32.8

**Table 2:** Content of potentially toxic elements in the samples of Technosol.

Sample	As mg/kg	Cd mg kg	Cu mg kg	Mn mg kg	Pb mg kg	Zn mg kg
1	511	8.76	8186	2647	2964	25,107
2	509	8.6	9227	2670	3387	24,786
3	343	12.7	7980	2417	4577	37,818
4	305	12.1	8336	2648	5078	47,291
5	318	13.4	7372	2425	4403	37,741

**Table 3:** Assignment of bacterial 16S rDNA soil isolates to the closest identified match in the GenBank database and their occurrence in the investigated samples of Technosol.

Soil isolates^a^	Species affiliation^b^	Samples				
		1^c^	2^c^	3^c^	4^c^	5^c^
SL – 2 (1) [KC817535]	*Pseudomonas* sp. B7 (2009)[FJ605431], 99% (γ-*Proteobacteria*)	+	–	–	–	–
SL – 5 (3) [KC817536]	*Variovorax paradoxus*, DSM 30162 [AB622223], 86% (β-*Proteobacteria*)	–	–	+	–	–
SL – 1 (2) [KC817534]	*Staphylococcus pasteuri*, Sp-12 [HM130543], 99% (*Firmicutes*)	–	–	+	–	+
SL – 6 (1) [KC817537]	Uncultured bacterium clone FWB10-MS [JQ480750], 92% (*Firmicutes*)	–	–	–	–	+
SL – 11 (2) [KC817538]	Uncultured bacterium clone ELU0126-T312-S-NI-000133 [HQ792208], 97% (*Actinobacteria*)	–	+	+	–	–
MR-1 (1) [JQ965935]	*Bacillus* sp. BA-27 [HF678939], 100% (*Firmicutes*)	–	+	–	–	–
MR-4 (2) [JQ965938]	*Bacillus* sp. BA-113 [HF678926], 99% (*Firmicutes*)	–	+	–	–	–
MR-2 (1) [JQ965936]	*Arthrobacter* sp. A8 [JX010953], 100% (*Actinobacteria*)	–	+	–	–	–
MR-3 (1) [JQ965937]	*Kocuria* sp. TMT4-12-2 [JX949820], 99% (*Actinobacteria*)	–	+	–	–	–

a Numbers in round brackets indicate the frequency of isolate occurrences in the soil samples, and numbers in square brackets indicate the GenBank accession number of identified 16S rDNA.

b Species assignment of bacterial 16S rDNA to the closest identified match in the GenBank database. Numbers in square brackets indicate the GenBank accession number and the level of identity is shown after the clone designation, and in round brackets, there is depicted the species assignment of bacterial 16S rDNA to the phylum.

c Depth of sampling: 1 and 3 = 0–10 cm; 2 and 4 = 20–30 cm; 5 = 0–2 cm.

**Table 4:** Microscopic fungi isolated from the investigated samples of Technosol.

Identification on the basis of the highest ITS similarity score	Samples				
	1^a^	2^a^	3^a^	4^a^	5^a^
JN596334.1*Acremonium furcatum* 99%	–	–	+	–	–
JQ316527*Aspergillus clavatus* 100%	–	–	–	–	+
KF031033.1*Aspergillus niger* 100%	+	+	+	+	+
*Aspergillus flavus*	–	–	–	–	+
KC461554.1*Aspergillus versicolor* 100%	–	+	–	–	–
JN226986*Aspergillus* sp. 100%	–	+	–	–	+
FJ608590.1*Bjerkandera adustata* 99%	+	–	–	–	–
FJ025204.1*Bionectria ochroleuca* 99%	–	–	+	–	+
KC806265.1*Clonostachys pseudochroleuca* 99%	–	–	+	–	–
FN539064.1*Lewia infectoria* 100%	–	–	–	–	+
JQ988826.1*Mortierella alpina* 99%	–	–	+	–	–
AJ301993.1*Myrothecium roridum* 99%	+	–	–	–	+
AF034463*Penicillium canescens* 99%	–	–	–	–	+
JF807949.1*Penicillium chrysogenum* 100%	–	+	–	+	–
DQ888735.1*Penicillium spinulosum* 99%	+	–	–	–	–
HQ443258.1*Penicillium* sp. 100%	–	–	–	+	–
FM999988.1*Phialocephala* sp. 100%	–	–	–	–	+
DQ474115.1*Phoma macrostoma* 99%	–	–	–	–	+
DQ117961.1*Rhizoctonia* sp. 100%	–	+	–	–	–
*Trichoderma* sp.	+	+	–	–	–

a Depth of sampling: 1 and 3 = 0–10 cm; 2 and 4 = 20–30 cm; sample 5 = 0–2 cm.

**Table 5:** Similarity (S) and dissimilarity (D) of mycocoenoses according to Jaccard (S_J_) and Sörensen (S_S_) in % of the investigated samples of Technosol.

Locality	S_J_ %	D %	S_S_ %	D %
1–2	15.38	84.62	26.66	73.34
1–3	9.09	90.91	16.66	83.34
1–4	11.11	88.89	20	80
1–5	11.76	88.24	21.05	78.95
2–3	8.33	91.67	15.38	84.62
2–4	18.18	81.82	30.76	69.24
2–5	11.11	88.89	20	80
3–4	11.11	88.89	20	80
3–5	11.76	88.24	21.05	78.95
4–5	7.14	92.86	13.33	86.67
